# Prevalence and associated risk factors of uterine fibroids in the Kingdom of Eswatini

**DOI:** 10.4102/safp.v68i1.6288

**Published:** 2026-07-17

**Authors:** Vuyisile J. Ginindza, Makandwe Nyirenda, Ropo E. Ogunsakin, Themba G. Ginindza

**Affiliations:** 1 Discipline of Nursing, College of Health Sciences, University of KwaZulu-Natal, Durban, South Africa; 2 Department of Public Health Medicine, Discipline of Nursing, College of Health Sciences, University of KwaZulu-Natal, Durban, South Africa; 3 Burden of Disease Research Unit, South African Medical Research Council, Cape Town, South Africa; 4 Department of Medicine, Epidemiology and Biostatistics, School of Medicine and Dentistry, Western University, Ontario, Canada

**Keywords:** uterine fibroids, prevalence, hysterectomy, myomectomy, risk-associated factors, Eswatini

## Abstract

**Background:**

The global burden of uterine fibroids (UFs) has increased from 4.5% to 68.6%, but insufficient data in sub-Saharan Africa limit the development of effective prevention and treatment strategies. The study aimed to determine the prevalence and associated risk factors of symptomatic UFs in Eswatini.

**Methods:**

An analytical cross-sectional study used purposive sampling to enrol 645 women aged 25–64 years old with UFs or symptoms. Data were collected via face-to-face interviews with a standardised questionnaire and analysed with descriptive statistics and logistic regression.

**Results:**

The overall prevalence of UFs was 67.8% (437/645). A higher prevalence was evident in the age group 50 years and older (81.4%) compared to prevalence (33.3%) in the age group 25–29 years. Notably, high prevalence was among people with diabetes (88.5%), widows (80%), those with a primary level of education (79.1%), the obese (76.3%), Depo-Provera (75.6%) and Hhohho (76.3%) participants. The univariate analysis significant association show that: for (1) ages 30–34 years (OR: 2.33; 95% CI: 1.26–4.35, *p* < 0.007), 35–39 years (OR: 5.08; 95% CI: 2.82–9.36, *p* < 0.001), 40–44 years (OR: 7.77, 95% CI: 4.19–14.9, *p* < 0.001), 45–49 years (OR: 5.50; 95% CI: 2.96–10.5, *p* < 0.001) and 50+ years (OR: 8.80, 95% CI: 4.58–17.6, *p* < 0.001); (2) primary education (OR: 2.43; 95% CI: 1.27–4.84, *p* = 0.009), and secondary education (OR: 1.88; 95% CI: 1.12–3.18, *p* = 0.017); (3) been pregnant (OR: 1.53; 95% CI: 1.04–2.23, *p* = 0.029); (4) obesity (OR: 3.39, 95% CI:2.02–5.70, *p* < 0.001); and (5) overweight (OR: 1.71; 95% CI: 1.02–2.89, *p* = 0.044). After adjustments, age remained significantly associated.

**Conclusion:**

Uterine fibroids are highly prevalent and significantly associated with age, underscoring the need for targeted awareness and future research.

**Contribution:**

The study provided insights to inform policy and enhance preventive strategies within the sexual reproductive health programs.

## Introduction

Uterine fibroids (UFs), referred to as uterine leiomyomas, are noncancerous smooth muscle tumours of the uterus, most common among women in the reproductive ages.^1^ Previous studies identify at least 12 risk factors.^1^ The global burden of UFs, as measured by disability-adjusted life years (DALYs), indicated a noticeable increase in the disease between 1990 and 2019.^2^ The prevalence of UFs generally differs by data collection methodology and setting.^3^ An international internet-based survey of 21,746 women from Brazil, Canada, France, Germany, Italy, South Korea, the UK and the USA reported a low prevalence of 9.8% in the UK and a high of 17.8% in Italy.^4^ In Canada, the prevalence of UFs was estimated to affect 70% of women by age 50; in Slovenia, 21.1% of women aged 30–55 years were most affected.^5,6^

A research study from China indicated that the prevalence of UFs increased from 48.4% in 1990 to 60.7% in 2016.^7^ The study further found that only females over age 15 were affected, and the most affected age group was women aged 45–49 years.^7^ Other associated risk factors identified in a similar study from China were early menarche, having children at an early age, low parity and a variety of reproductive diseases, namely vaginitis, endometriosis, ovarian cysts and menstrual disorders.^8^ The worldwide incidence and prevalence of UFs have been increasing in the past decade, positively correlated with alcohol intake, hypertension, overweight and obesity.^9^ Additionally, the age-specific incidence and prevalence of UFs in most age groups showed increasing trends except for 45–54-year-old women.^10^ Moreover, countries like those in Eastern Europe, Tropical Latin America, Brazil and India experience the greatest uterine fibroid burden.^11^ Globally, women aged 35–39 years and older indicated an increased risk of UFs, as reflected in the higher incidence rates among these age groups.^11,12,13^ A recent systematic review of the literature indicated that the prevalence of UFs was consistently higher in black than in white women.^14^ Recent research studies also highlighted other risk-associated factors of UFs, including current alcohol intake, chronic psychological stress, earlier menstrual age, hypertension, obesity and elevated 2-h glucose post-challenge.^15,16^ Uterine fibroids-associated risk factors vary from population to population; in addition to black race, age and high body mass index (BMI) were often among the strongest UFs risk factors.^1,17,18^ Research findings from the African continent indicated that overweight, obesity and older reproductive age increase the risk of UFs among women.^19,20,21,22,23^ Previous studies found a positive association between hypertension and UFs.^24,25,26^ Furthermore, participants not taking hypertension treatment or those with new-onset hypertension indicated a high risk of developing UFs.^27^ Family history of UFs indicated an association with the incidence of UFs; maternal history of UFs increased the risk of developing new fibroids in black African and American women.^28^

The African continent has fewer research studies on UFs than other regions.^29^ A study conducted in Nigeria indicated that UFs were more prevalent among women aged 30–44 years (75.6%) and less prevalent in women aged 20–24 years and 50–54 years (1.9%).^30^ Nulliparous women were the leading group at risk of developing UFs (77.7%).^30^ Uterine fibroids have been noted to be the major cause of hysterectomies,^31^ and 15.3% of women who had undergone a caesarean section had myomas.^32^ In other countries like Côte d’Ivoire and Ghana, UFs were associated with infertility.^19,33,34^ Findings from another study conducted in Nigeria indicated similar UFs’ risk associated with those identified by other researchers globally: hypertension, diabetes mellitus, smoking, infertility, high BMI and reproductive-age women.^35,36,37^ Meanwhile, in Ghana, monosodium glutamate (MSG), found in almost all food colourants on Ghanaian markets, was identified as a risk factor for the development of UFs.^38^

The epidemiology of UFs (also known as myomas) remains a mystery; more research on the associated risk factors for UFs to improve treatment measures, such as developing non-surgical treatment options for myomas, has been suggested.^39^ It is critical to examine risk-associated factors in symptomatic women with UFs to understand other ethnic factors, as cultural and familial factors have been identified to have a significant impact on uterine fibroid diagnosis and management.^40^ Furthermore, understanding non-genetic risk factors and intervention drivers can improve equity and reduce vulnerability and poor outcomes in the management of UFs, especially among high-risk women who face high treatment expectations and financial obstacles.^41,42,43^ There is limited epidemiological data on UFs from sub-Saharan African (SSA) countries such as Eswatini. As a result, there are no clear guidelines for planning and designing health strategies to prevent, treat and manage UFs. This study, therefore, aimed to determine the prevalence of UFs and their associated risk factors among women attending selected health facilities in Eswatini.

## Research methods and design

### Study design

An analytical cross-sectional study was conducted across eight study sites to collect data on socio-demographics, sexual and reproductive health, the prevalence of UFs and associated risk factors among women seeking health services at gynaecology outpatient departments or admitted to gynaecological wards. Women with signs or symptoms associated with UFs (abnormal menstrual bleeding, heavy, prolonged bleeding), pelvic pain, pelvic pressure, enlarged abdomen, lower abdominal pain and back pain or ultrasound-confirmed or unconfirmed UFs diagnoses or those who had undergone UFs-related surgery (hysterectomy, myomectomy, laparoscopic procedure or uterine embolisation) were targeted. To determine the prevalence of a disease, one requires a study population of both patients with the disease and those without.^44,45^

### Study setting

This study was conducted in the Kingdom of Eswatini, a sovereign state in Southern Africa bordered by South Africa and Mozambique. It is one of Africa’s smallest countries and is classified as a lower-middle-income country. Eswatini has four geographical regions: Hhohho, Manzini, Lubombo and Shiselweni, with an estimated population of 1.2 million. Human development is medium, according to the 2022 Human Development Index (0.60). The study was conducted within a hospital-based sample of women from the four geographic regions between 21 August 2021 and 21 August 2022. The women were recruited from eight study sites, namely Piggs Peak Government Hospital, Mbabane Government Hospital, Mbabane Private Clinic, Raleigh Fitkin Memorial Hospital, Women and Children Private Clinic, Mankayane Government Hospital, Hlatikulu Government Hospital and Good Shepherd Mission Hospital, representative of the country’s main hospitals.

### Sample size

To estimate the prevalence of UFs, three considerations informed the optimal sample size for this study: a 95% desired confidence interval, an accepted 5% margin of error and the estimated prevalence of the outcome of interest. The prevalence of the outcome of interest was assumed unknown, with a maximum variability of 50%. A sample size of 384 participants was required; this was increased by 13% to account for potential rejections, then multiplied by a design effect (D) of 1.5, yielding a final desired sample size of 652 women. However, 645 women were eventually enrolled, as some screened women did not meet the age criterion for inclusion. Our approach to determining the required sample size was in line with established guidelines for health research, as sample size calculations are directly related to the variance. Assuming a 50% prevalence yields the most conservative estimate for the sample size, meaning the largest sample size is obtained. This approach minimises the risk of underestimating the required sample size and ensures adequate statistical power for the study.^46,47^ Increasing the sample size reduced the type I and type II errors, as well as the effects of known and unknown confounders. The power (1-β) (the % chance of detecting a difference) of the study was set at 80%.^48^

### Sampling method

Admitting hospitals across the four Eswatini regions were stratified by facility type (government, mission or private). From these, eight hospitals were purposively selected based on the presence of a gynaecologist, a radiology department with fully functioning equipment (e.g. scans) and an obstetric department. Participants were recruited using purposive sampling. Women attending the gynaecology outpatient departments or admitted to the wards were screened and purposively enrolled as they presented to the gynaecology clinics, provided they met the inclusion criteria. These included women aged 25–64 years with signs and symptoms of UFs (pelvic pain, abnormal menstruation), an unconfirmed or confirmed UFs diagnosis or a history of any UFs-related surgical procedure (hysterectomy, myomectomy, laparoscopic or uterine embolisation), who had voluntarily consented. Women younger than 25 years or older than 64 years, without a confirmed UFs diagnosis or UFs-related surgery and not attending the gynaecology outpatient departments or admitted to the wards were excluded from the study. Enrolment was offered equally to participants, irrespective of culture, religion, race and social class. Trained research assistants (RAs) individually explained the study aims and procedures to women willing to participate. We also discussed the potential risks and benefits of participating in the study with each participant before obtaining their written consent. To protect their privacy, we assigned them an identification number unrelated to their real names and surnames.

### Data collection

Data were collected through face-to-face interviews using structured questionnaires. A pilot study was conducted at Manzini Clinic, which was not among the selected study sites. The pilot informed refinements to the tools and additional training for the RAs, who were registered nurses. The RAs administered the questionnaires to participants to collect sociodemographic data (age, marital status, nature of work); complete health history; history of chronic diseases such as diabetes mellitus and hypertension; family history of UFs; lifestyle history (exercise, alcohol use and smoking) and clinical presentation data. All participants underwent a pelvic ultrasound scan to confirm UF diagnoses. Each participant was assigned a unique study identification number to link the questionnaire to the electronic database and prevent data errors. Data were then entered into Epi Info software (7.2.2.5) and stored in a central location. Access to data was restricted to the data manager and the principal investigator to ensure privacy, safety and confidentiality.

### Data analysis

#### Statistical analyses

Data were exported from Epi Info software (7.2.2.5) (Stata Corporation, College Station, Texas, USA) to Stata 18.5 (Stata Corporation, College Station, Texas, USA), cleaned and checked for possible errors and missing values before analysis.^49^ Questionnaires with missing values were thoroughly assessed to determine whether they affected the research outcomes before being excluded from the analysis. The data are presented as the mean with standard deviation (s.d.) for normally distributed continuous variables and as the median with interquartile range (IQR) for skewed continuous variables. Categorical data are presented as absolute and relative frequencies (*n* and %). Baseline characteristics were compared between the outcome of interest and the explanatory variables using the chi-square test. Odds ratios are reported as point estimates with 95% CIs, and *p* < 0.05 is considered significant. All statistical analyses were performed using STATA.

### Ethical considerations

Before data collection, ethical approval was obtained from the University of KwaZulu-Natal Biomedical Research Ethics Committee (BREC) (BREC/00002571/2021) and the Eswatini Health and Human Research Review Board (EHHRRB023/2021). Permission was also obtained from the study site gatekeepers. After eligible participants were identified, the research aims and objectives were explained to them individually with assistance from the RAs and registered nurses. Potential participants were given time to read and understand the consent form. They were encouraged to ask questions, and both written and verbal consent were obtained. Participation in the research study was completely voluntary. Each participant was assigned a unique number to guarantee their anonymity.

## Results

### Characteristics of the study participants

Table 1 summarises the characteristics of the study participants. A total of 645 women participated in the study from August 2021 to December 2022. The mean age (± standard deviation) was 40.4 years (± 8.9); weight, 78.9 kg (± 13.6); height, 2.10 cm (± 8.9) and body mass index (BMI), 30.9 (± 5.13). Among the study participants, 20.3% were aged 35–39 years, 36.4% were from the Manzini region, and 57.8% resided in rural areas. Approximately 47.9% of the participants were employed, and 47% had tertiary-level education, which is not representative of educational trends in Eswatini. This could be influenced by the purposive sampling method used in the study and other socioeconomic factors. More than half of the participants were married (56.3%), and 76.9% had experienced at least one pregnancy. About 90% of participants menstruated before the age of 15, 35% used the Depo Provera (DMPA) contraceptive method, 51.6% were classified as obese and 36.6% were categorised as overweight. Some participants had chronic diseases; 22.5% were hypertensive, and 21.9% had a positive human immunodeficiency virus (HIV) status.

**TABLE 1:** Socio-demographic characteristics of enrolled participants (*N* = 645).

Demographics	*n*	%	Mean	s.d.
**Overall prevalence**	645		40.38	8.9476
No	208	32.2	-	-
Yes	437	67.8	-	-
**Age groups (years)**				
25–29	81	12.6	-	-
30–34	93	14.4	-	-
35–39	131	20.3	-	-
40–44	127	19.7	-	-
45–49	105	16.3	-	-
≥ 50	108	16.7	-	-
**Marital status**				
Married	363	56.3	-	-
Single	16	2.5	-	-
Unmarried	216	33.5	-	-
Widowed	50	7.8	-	-
**Education**				
High school	136	21.1	-	-
Primary	72	11.2	-	-
Secondary	134	20.8	-	-
Tertiary	303	47.0	-	-
**Region**				
Hhohho	186	28.8	-	-
Lubombo	114	17.7	-	-
Manzini	235	36.4	-	-
Shiselweni	110	17.1	-	-
**Residence**				
Rural	373	57.8	-	-
Urban	272	42.2	-	-
**Employment status**				
Employed	309	47.9	-	-
Self-employed	79	12.2	-	-
Unemployed	257	39.8	-	-
**Family history of uterine fibroids**				
No	354	54.9	-	-
Yes	291	45.1	-	-
**Alcohol**				
No	485	75.2	-	-
Yes	160	24.8	-	-
**Cigarette smoking**				
No	606	94.0	-	-
Yes	39	6.0	-	-
**Exercise**				
No	457	70.9	-	-
Yes	188	29.1	-	-
**Caffeine consumption**				
No	109	16.9	-	-
Yes	566	83.1	-	-
**Age of menarche (years)**				
< 15	581	90.1	-	-
> 15	64	9.9	-	-
**Ever been pregnant**				
No	149	23.1	-	-
Yes	496	76.9	-	-
**Current use of contraceptives**				
No	511	79.2	-	-
Yes	134	20.8	-	-
**Types of contraceptives ever used**				
Bilateral tubal litigation	4	0.6	-	-
Combined oral pills	104	16.1	-	-
Depo Provera	226	35.0	-	-
Never used contraceptives	157	24.3	-	-
Non-hormonal	84	13.0	-	-
Norplant	70	10.9	-	-
**History of sexually transmitted disease infections**				
Genital warts	34	5.3	-	-
Gonorrhoea	90	14.0	-	-
Syphilis	66	10.2	-	-
None	455	70.5	-	-
Weight mean	78.9	13.64	-	-
Height mean	2.10	8.99	-	-
BMI mean	30.91	5.13	-	-
**Current BMI**				
Normal weight	76	11.8	-	-
Overweight	236	36.6	-	-
Obese	331	51.6	-	-
**Chronic diseases**				
Diabetes mellitus	26	4.0	-	-
HIV	141	21.9	-	-
Hypertension	145	22.5	-	-
Cancer of the cervix	22	3.4	-	-
None	311	48.2	-	-

BMI, body mass index, s.d., standard deviation; HIV, human immunodeficiency virus.

### Overall prevalence of uterine fibroids

Table 2 shows the prevalence of symptomatic UFs in Eswatini. The hospital-based, symptom-defined prevalence of UFs is calculated as the number of participants with confirmed UFs at a given time divided by the total number of participants in the sample.

**TABLE 2:** The overall uterine fibroids prevalence (*N* = 645).

Classifications	Uterine fibroid				*p*
	*N*	*n*	%	95% CI	
**Overall prevalence**	645	437	67.8	61.6–74.4	
**Age groups**					**< 0.001**
25–29	81	27	33.3	21.9–48.5	-
30–34	93	50	53.8	39.9–70.9	-
35–39	131	94	71.8	57.9–87.8	-
40–44	127	101	79.5	64.8–96.6	-
45–49	105	77	73.3	57.9–91.7	-
≥ 50	108	88	81.4	63.4–100.0	-
**Marital status**					**0.002**
Married	363	260	71.6	63.1–80.9	-
Single	16	11	68.8	34.3–100.0	-
Unmarried	216	126	58.3	48.6–69.5	-
Widowed	50	40	80.0	57.2–100.0	-
**Level of education**					**0.011**
High school	136	83	61.0	48.6–75.7	-
Primary	72	57	79.2	59.9–100.0	-
Secondary	134	100	74.6	60.7–90.8	-
Tertiary	303	197	65.0	56.3–74.8	-
**Region**					**0.001**
Hhohho	186	142	76.3	64.3–89.9	-
Lubombo	114	65	57.0	44.0–72.7	-
Manzini	235	164	69.8	59.5–81.3	-
Shiselweni	110	66	60.0	46.4–76.3	-
**Residence**					0.472
Rural	373	248	65.0	58.5–76.3	-
Urban	272	189	66.5	59.9–80.1	-
**Employment status**					0.367
Employed	309	201	65.1	56.4–74.7	-
Self-employed	79	56	70.9	53.5–92.1	-
Unemployed	257	180	70.1	60.2–81.1	-
**Family history of uterine fibroids**					0.820
No	354	238	67.2	58.9–76.3	-
Yes	291	199	68.4	59.2–78.6	-
**Alcohol consumption**					0.142
No	485	329	67.8	60.7–75.6	-
Yes	160	128	67.5	55.4–81.5	-
**Cigarette smoking**					0.978
No	606	410	67.7	61.3–74.5	
Yes	39	27	69.2	45.6–100.0	
**Exercise**					0.276
No	457	316	69.2	61.7–77.2	-
Yes	188	121	64.4	53.4–76.9	-
**Caffeine consumption**					0.937
No	109	73	67.0	52.5–84.2	-
Yes	536	364	68.0	61.1–75.3	-
**Age of menarche (years)**					0.376
< 15	581	390	67.1	60.6–74.1	-
> 15	64	47	73.4	53.9–97.7	-
**Ever been pregnant**					**0.037**
No	149	90	60.4	48.6–74.2	-
Yes	496	347	70.0	62.8–77.7	-
**Current use of contraceptives**					**0.033**
No	511	357	70.0	62.8–77.5	-
Yes	134	80	59.7	47.3–74.3	-
**Types of contraceptives used**					**< 0.001**
Bilateral tubal ligation	4	3	75.0	15.5–21.9	-
Combined oral pills	104	76	73.1	57.6–91.5	-
Depo Provera	226	171	75.7	64.7–87.9	-
Never used contraceptives	157	103	65.6	56.4–83.1	-
Non-hormonal	84	53	63.1	47.3–82.5	-
Norplant	70	31	44.3	30.1–62.9	-
**Sexually transmitted infections**					0.419
Genital warts	34	26	76.4	5.0–11.2	-
Gonorrhoea	90	63	70.0	53.8–89.6	-
Syphilis	66	48	72.7	53.6–96.4	-
None	455	300	65.9	58.6–73.8	-
**Current BMI**					**< 0.001**
Normal weight	76	37	48.7	34.3–67.1	-
Obesity	333	254	76.3	67.2–86.3	-
Overweight	236	146	61.9	52.2–72.8	-
**Chronic diseases**					**< 0.001**
Cancer of the cervix	22	18	81.8	49.0–100.0	-
Diabetes mellitus	26	23	88.5	56.0–100.0	-
HIV	141	101	71.6	58.3–87.0	-
Hypertension	145	109	75.2	61.7–90.7	-
None	311	186	59.8	51.5–69.0	-

Note: The bold values signify all the variables that were associated to uterine fibroids, with *p*-values less than 0.05.

CI, confidence interval; BMI, body mass index; HIV, human immunodeficiency virus.

**FIGURE 1:**
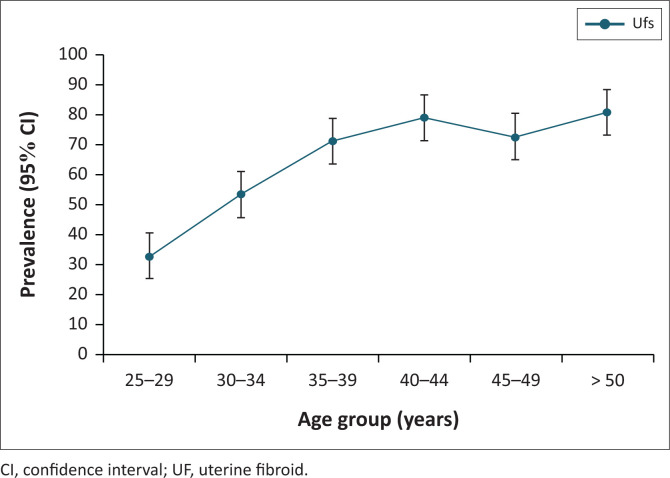
Age-specific prevalence of uterine fibroids among women in Eswatini.

The overall prevalence of UFs was 67.8% (95% CI: 61.5–74.4). The prevalence among the women with symptomatic UFs increased with age: from 33.3% (95% CI: 21.9–48.5) among women aged 25–29 years to 53.8% (95% CI: 39.9–70.9) for those aged 30–34 years, 71.8% (95% CI: 57.9–87.8) for those aged 35–39 years and 79.5% (95% CI: 64.8–96.6) for those aged 40–44 years, respectively (Figure 1). The prevalence then declined for those aged 45–49 years to 73.3% (95% CI: 57.9–91.7) and thereafter increased to 81.4% (95% CI: 63.4–100.3) for those aged 50 years. The prevalence was significantly higher among women aged 50 years and older than among women aged 25–29 years, as shown in Figure 1.

Those with a primary level of education had a higher observed prevalence of UFs, 79.2% (95% CI: 59.9–102.6), than those with secondary education, 74.6% (95% CI: 60.7–90.8) (Table 2). The observed prevalence of UFs for participants residing in the Hhohho (76.3%, 95% CI 64.3–89.9) and Manzini regions (69.8%, 95% CI: 59.5–81.3) was higher than that of those who reside in Lubombo (57.0%, 95% CI: 44.0–72.7) and Shiselweni (60.0%, 95% CI: 46.4–76.3; Table 2). The observed prevalence of UFs was high across all four chronic diseases among the women with confirmed UF diagnoses, ranging from 75.2% to 88.5% (hypertension: 75.2%, 95% CI: 61.7–90.7; diabetes mellitus: 88.5%, 95% CI: 56.1–132.7 and positive HIV status: 71.6%, 95% CI: 58.3–87.0), as shown in Table 2. The prevalence was higher among participants who were not currently using any form of contraception (70%, 95% CI: 62.8–77.5) than among those who were currently using contraceptives (59.7%, 95% CI: 43.7–74.3). The prevalence was noticeably higher among participants who used Depo-Provera (75.7%, 95% CI: 64.7–87.9) than among those using other contraceptive methods (combined oral contraceptives: 73.1%, 95% CI: 57.6–91.5; Norplant: 44.3%, 95% CI: 30.1–62.9).

There was a higher prevalence among participants who had been pregnant in their lifetime at 70.0% (95% CI: 62.8–77.7). According to the marital status of the women diagnosed with UFs, the prevalence was higher among widowed and married participants: 80.0% (95% CI: 62.8–77.7) and 71.6% (95% CI: 63.1–80.9), respectively. The observed prevalence of UFs was higher among obese and overweight participants, 76.3% (95% CI: 67.2–86.3) and 61.9% (95% CI: 52.2–72.8), respectively.

### Risk factors associated with uterine fibroids

Table 3 summarises the risk factors associated with UFs. All covariates were included in the multivariate analysis: those that were significant and those that lost significance in the univariate analysis. However, all variables that lost significance in the univariate analysis have been removed, and only significant socio-demographic variables have been presented in the table. The research methodology used in this study emphasised including numerous risk-related factors, utilised a large sample size and employed logistic regression analysis to account for multiple confounders.^48^ Age, educational status, ever being pregnant and BMI were associated with UFs in univariate analyses (Table 3). Based on the univariate analysis, ages 30–34 years (OR: 2.33; 95% CI: 1.26–4.35, *p* < 0.007), 35–39 years (OR: 5.08; 95% CI: 2.82–9.36, *p* < 0.001), 40–44 years (OR: 7.77; 95% CI: 4.19–14.9, *p* < 0.001), 45–49 years (OR: 5.50; 95% CI: 2.96–10.5, *p* < 0.001), 50+ (OR: 8.80; 95% CI: 4.58–17.6, *p* < 0.001), primary level of education (OR: 2.43; 95% CI: 1.27–4.84, *p* = 0.009), secondary level of education (OR: 1.88; 95% CI: 1.12–3.18, *p* = 0.017), ever been pregnant (OR: 1.53; 95% CI: 1.04–2.23, *p* = 0.029) and BMI (obesity (OR: 3.39, 95% CI: 2.02–5.70, *p* < 0.001), overweight (OR: 1.71; 95% CI: 1.02–2.89, *p* = 0.044) were statistically significantly associated with UFs (Table 3). However, alcohol consumption (OR: 0.98; 95% CI: 0.67–1.45, *p* = 0.937), exercise (OR: 0.81; 95% CI: 0.56–1.16, *p* = 0.238), sexually transmitted infections (gonorrhoea: OR: 0.72, 95% CI: 0.27–1.73, *p* = 0.476 and syphilis: OR: 0.82, 95% CI: 0.30–2.10, *p* = 0.686), Manzini region (OR: 0.72; 95% CI: 0.46–1.11, *p* = 0.135), marital status (single: OR: 0.87, 95% CI: 0.31–2.82, *p* = 0.803), combined oral contraceptives pills (OR: 0.90; 95% CI: 0.04–7.40, *p* = 0.932), Norplant (OR: 0.26; 95% CI: 0.01–2.18, *p* = 0.260), non-hormonal contraceptive methods (OR: 0.57; 95% CI: 0.03–467, *p* = 0.633), hypertension (OR: 0.67; 95% CI: 0.19–1.95, *p* = 0.498), HIV (OR: 0.56; 95% CI: 0.15–1.62, *p* = 0.322), tertiary level of education (OR:1.19; 95% CI: 0.78–1.80, *p* = 0.422), diabetes mellitus (OR: 1.70; 95% CI: 0.33–9.59, *p* = 0.519), being widowed (OR: 1.58; 95% CI:0.79–3.46, *p* = 0.26), urban residence (OR: 1.15; 95% CI: 0.82–1.61, *p* = 0.421), self-employed (OR: 1.31; 95% CI: 0,77–2.28, *p* = 0.328), employed (OR: 1.26; 95% CI: 0.88–1.79, *p* = 0.208) and age of menarche (OR: 1.35; 95% CI: 0.77–2.48, *p* = 0.307) (Table 3) were inversely associated with UFs and not statistically significant.

**TABLE 3:** Risk factors associated with uterine fibroids (*N* = 642).

Risk factors	Odds ratio	95% CI	*p*	Adjusted odds ratio	95% CI	*p*
**Age groups (years)**						
25–29	-	-	-	-	-	-
30–34	2.33	1.26–4.35	< 0.007	1.81	0.90–3.68	0.100
35–39	5.08	2.82–9.36	**< 0.001**	4.42	2.16–9.28	**< 0.001**
40–44	7.77	4.19–14.9	**< 0.001**	7.86	3.52–18.2	**< 0.001**
45–49	5.50	2.96–10.5	**< 0.001**	6.14	2.55–15.2	**< 0.001**
≥ 50	8.80	4.58–17.6	**< 0.001**	11.3	4.06–32.8	**< 0.001**
**Marital status**						
Married	-	-	-	-	-	-
Single	0.87	0.31–2.82	0.803	1.06	0.33–4.36	0.818
Unmarried	0.55	0.39–0.79	**0.001**	1.06	0.67–1.68	0.818
Widowed	1.58	0.79–3.46	0.26	1.15	0.47–2.94	0.763
**Region**						
Hhohho	-	-	-	-	-	-
Lubombo	0.41	0.25–0.68	**< 0.001**	0.28	0.15–0.53	**< 0.001**
Manzini	0.72	0.46–1.11	0.135	0.72	0.42–1.23	0.232
Shiselweni	0.46	0.28–0.77	**< 0.003**	0.41	0.21–0.79	**0.007**
**Residence**						
Rural	-	-	-	-	-	-
Urban	1.15	0.82–1.61	0.421	1.54	0.98–2.44	0.062
**Educational level**						
High school	-	-	-	-	-	-
Primary	2.43	1.27–4.84	**0.009**	1.52	0.70–3.41	0.294
Secondary	1.88	1.12–3.18	**0.017**	1.59	0.87–2.93	0.134
Tertiary	1.19	0.78–1.80	0.422	1.26	0.74–2.14	0.395
**Current contraceptive use**						
No	-	-	-	-	-	-
Yes	0.64	0.43–0.95	**0.026**	0.92	0.54–1.59	0.763
**BMI**						
Normal weight	-	-	-	-	-	-
Obesity	3.39	2.02–5.70	**< 0.001**	1.22	0.55–2.73	0.625
Overweight	1.71	1.02–2.89	**0.044**	1.34	0.70–2.56	0.374

Note: The bold values signify all the variables that were associated to uterine fibroids, with *p*-values less than 0.05.

CI, confidence interval; BMI, body mass index.

After the multivariable adjustment, ages 35–39 years (aOR: 4.42; 95% CI: 2.16–9.28, *p* < 0.001), 40–44 years (aOR: 7.86; 95% CI: 3.52–18.2, *p* < 0.001), 45–49 years (aOR: 6.14; 95% CI: 2.55–15.2, *p* < 0.001) and ≥ 50 years (aOR: 11.3; 95% CI: 4.06–32.8, *p* < 0.001) remained significantly associated with UFs while being from Lubombo and Shiselweni indicated a protective effect (OR = 0.28; 95% CI: 0.15–0.53; *p* < 0.001 and OR = 0.41; 95% CI: 0.21–0.79; *p* < 0.007, respectively). Risk factors such as alcohol consumption, caffeine consumption, cigarette smoking, exercise, marital status, region, residence, employment status, level of education, ever being pregnant, age of menarche, family history of UFs, sexually transmitted infections, chronic diseases and contraceptive use methods (Table 3) were inversely but not significantly associated with UFs.

## Discussion

This study aimed to determine the prevalence of UFs and their associated risk factors in Eswatini to inform policies and programmes on screening, care and management of UFs. The results of this study showed a high prevalence of UFs (67.8%), which is comparable to findings from other countries such as Germany (48.6%) and China (40%).^7,50,51^ A study in Germany indicated that approximately 40% of women develop myomas in their lifetime.^25^ However, the findings of this study revealed a higher prevalence as compared to similar studies in other SSA countries, such as Uganda (28.2%), Ghana (28.2%) and Nigeria (29.3%),^21,37,52^ because of different research methodologies used.^53^

In our study, the prevalence of UFs significantly increased with age, which is consistent with findings from other studies.^7,20,51,54^ A study on the epidemiology of UFs in Germany revealed an association between age and myomas, with prevalence increasing from 21.3% (30–35 years) to between 62% and 85% (45–50 years).^19^ The positive association between age and UFs could be explained by the fact that as women advance in age, there are imbalances in the sex hormones and changes in DNA repair capacity that predispose them to multiple ailments, including UFs.^55^

Participants with a higher BMI showed a high prevalence of UFs and a marginal association with UFs, according to this study. Similar findings from other research studies in India, Iraq, China and Ghana identified a high prevalence with a significant association between UFs and BMI.^19,56,57,58,59^ Wong et al.^60^ revealed that obesity disrupts progesterone regulation, which renders the endometrium prone to overstimulation from no oestrogenic antagonist for a long period, resulting in the development of UFs.^60^ The study also indicated that UFs were common among participants with hypertension. Though our findings showed no significant association between hypertension, perhaps due to our cross-sectional study design, other researchers have shown a significant association between hypertension and risk of UFs.^26,27^ The findings from this study also indicated a high prevalence of UFs among participants with diabetes mellitus. Though our findings showed an inverse association, previous research findings revealed that UFs risk may increase with insulin resistance and decrease with diabetes treatment (metformin and myomectomy).^61,62,63^ The study also revealed a high prevalence of UFs among participants who used Depo-Provera. Recent research findings revealed that progesterone is involved in the pathogenesis of UFs; hence, the use of progesterone may increase the risk of UFs development, which explains the high prevalence of UFs among participants who use Depo-Provera in the study.^64^ The study also showed a high prevalence of UFs among widowed participants as compared to the married participants. The findings were contrary to previous research findings, which identified an association between married women and an increased risk of UFs.^65,66^

We assumed that since Eswatini is heavily burdened with HIV, this would be strongly associated with the risk of UFs. However, we found a negative association between HIV and UFs. We concur with the latest research findings that mention limited research on the effects of antiretroviral therapy and UF treatment care.^67^ This was the first UF prevalence study in the country, and there are no similar studies that discuss the relationship between HIV and UFs. In addition, we specifically targeted women attending gynaecology outpatient departments or admitted to gynaecological wards and not those who may have been at the facility for HIV care.

Some of the risk factors in our study had a high prevalence of UFs but lost significance in the multivariable analysis because of various reasons, such as the small sample size, and factors, such as cancer and diabetes mellitus. Body mass index is another factor that has lost its significance because it is correlated; when related factors are added, it reduces its unique contribution.

### Strengths

Our study’s key strength was the use of primary data to determine study outcomes. This is the first study looking at the burden of UFs in the country; other researchers may generate hypotheses from the identified relationships for future research. Our study was able to provide local data to inform policymakers and strengthen SRH policy/guidelines to incorporate UFs into the Sexual Reproductive Health (SRH) programme.

### Limitations

The research was hospital-based in gynaecology departments; thus, some participants could have been missed in other healthcare settings or those who did not come to the hospital. The inclusion criteria focused on participants with signs and symptoms, with or without a confirmed UFs diagnosis, yet UFs are asymptomatic at times, so some could have been missed. This study also used self-reported data, which can lead to over- or underestimation of the prevalence. The methodology used to select covariates in the multivariate analysis has the potential to introduce bias. Hence, we are proposing future longitudinal and clinical studies, as this was the first study in the country on the prevalence and risk factors associated with UFs. The study was done during the coronavirus disease 2019 (COVID-19) pandemic. The restrictions on the movement of people and hospital admissions criteria may have biased the estimation of the UFs’ prevalence. This bias is nearly impossible to quantify and account for in the estimate.

### Implications and recommendations

This study indicated a high prevalence of UFs in the country. The findings indicated high prevalence among participants with a higher BMI, with chronic diseases such as hypertension, diabetes mellitus and HIV/acquired immunodeficiency syndrome, widows and use of certain contraceptives such as Depo-Provera.

In Eswatini’s health facilities, there are currently no standard guidelines for managing UFs. We therefore recommend that guidelines be formulated for the care and management of the disease. These guidelines should incorporate educational programmes on weight management strategies introduced in gynaecological clinics, communities and hospitals to reduce the risk of UFs. Additionally, they include programmes for the screening and monitoring of chronic diseases (blood pressure treatment, diabetes mellitus, HIV/AIDS) and care among women attending gynaecological clinics.

Further investigations are required to broadly explain the complex interplay of factors influencing fibroid formation and the precise relationship with marital status. Consequently, we advocate for forthcoming longitudinal studies that will incorporate the age at which contraceptives are initiated and the duration of their usage to elicit excellent results.

## Conclusion

This analytical cross-sectional symptomatic hospital-based study highlighted a significant prevalence of UFs among women, which requires a multidimensional set of interventions to reduce the burden of UFs in our population. The study provided essential information about UFs’ associated risk factors, such as age, BMI, previous pregnancies and level of education. These results add to the limited evidence of UFs and will contribute to policy development and planning prevention strategies for UFs. Additionally, our findings support the suggestion to incorporate lifestyle approaches into the existing screening programme as part of a comprehensive routine screening strategy. Finally, our findings provide epidemiological knowledge about the distribution of UFs. Such information is crucial to guide the future planning and resource allocation to the SRH program in Eswatini. Future longitudinal research studies are recommended to detail all the specific risk-associated factors of this study and the use of a modified disjunctive cause criterion in the analysis.
